# Supplementary material for: Antimicrobial Activities of Propolis in Poloxamer Based Topical Gels
Source: Pharmaceutics. 2021 Nov 26;13(12):2021. doi: 10.3390/pharmaceutics13122021 (PMC8706605; doi:10.3390/pharmaceutics13122021)
Supplement: Supplementary file 1 [file pharmaceutics-13-02021-s001.zip › pharmaceutics-1419308-supplementary.pdf]

pharmaceutics

Supplementary Materials: Antimicrobial Activities of Propolis in Poloxamer Based Topical Gels

SeongHyeon An, Eunmi Ban, In-Young Chung, You-Hee Cho and Aeri Kim

Supplement 1. Size distribution measurement of various solutions by DLS

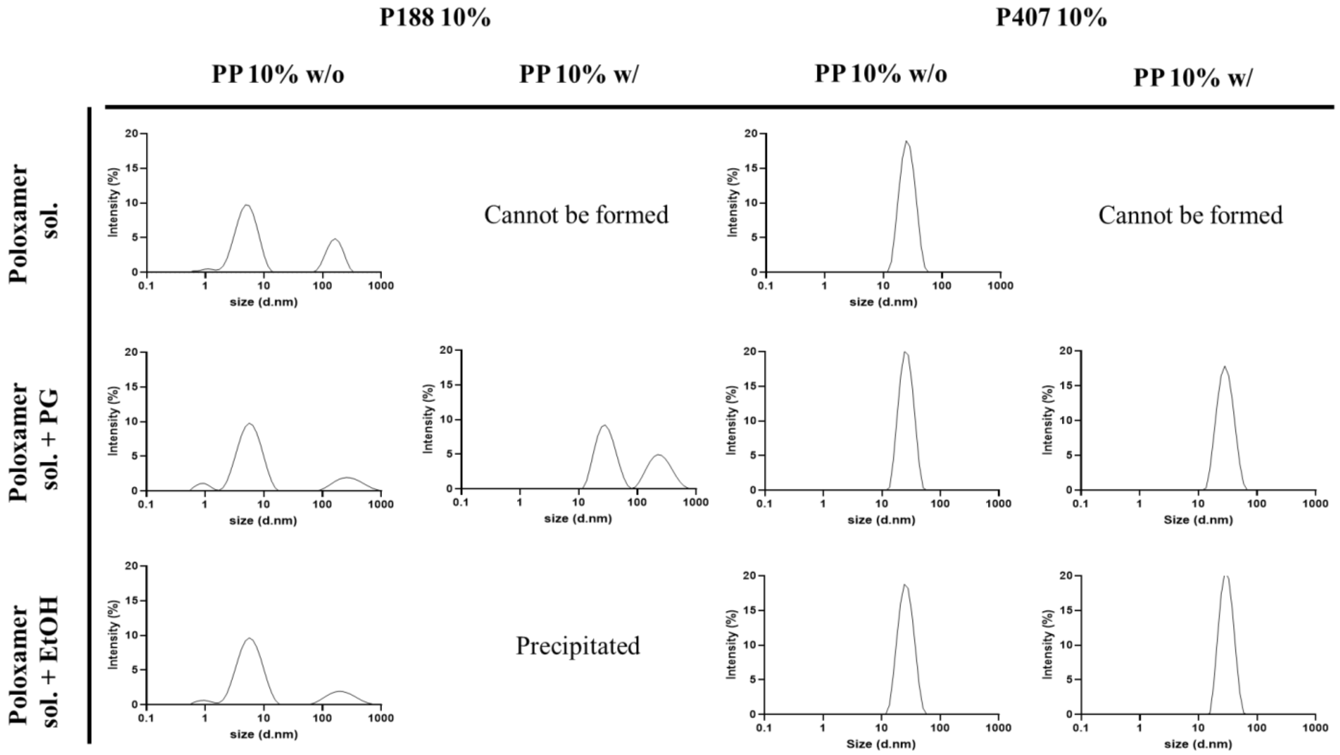

Figure S1. Representative size distribution profiles of various solutions obtained by DLS measurement. Poloxamer sol. + PG: poloxamer solution with propylene glycol; Poloxamer sol. + EtOH: poloxamer solution with ethanol; PP10% w/o: samples without propolis; PP10% w/: samples with 10% propolis extract.

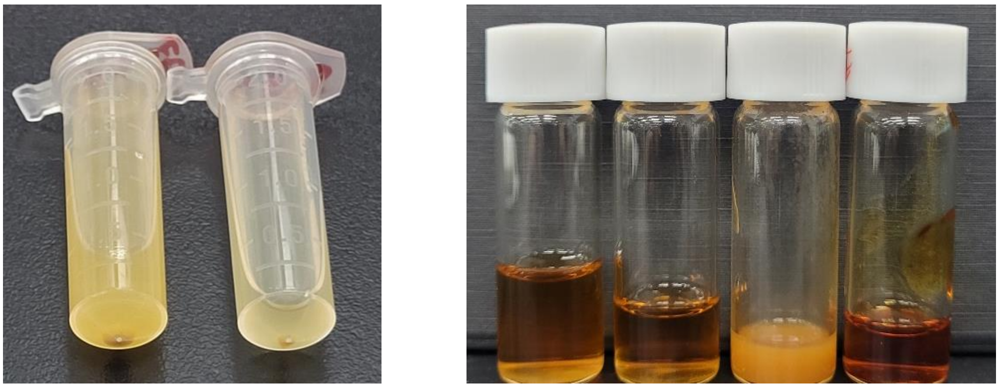

(**a**) (**b**)

Figure S2. Precipitates observed in P188 (**a**) 10% solution containing (**b**) 10% of propolis ethanol-extract.

Supplement 2. Rheological properties of poloxamer solutions
Experimental methods.

Preparation of propolis loaded poloxamer formulation

Propolis-loaded poloxamer solution was prepared according to the cold method. Briefly, P188 or P407 was first dissolved in cold water at room temperature (RT) under continuous slow agitation. After the completion of dissolution, propolis solution dissolved in PG was added to the poloxamer solution and then stirred. Propolis-loaded poloxamer solution was maintained at 4 °C prior to use.

Measurement of gelation temperature

The gelation temperature of propolis-loaded poloxamer formulation was measured using the tube invert method. Vials containing 1 g of poloxamer formulations left in an ice water bath. Vials were transferred from an ice-water bath to a water bath of RT or 37 °C. Then Vials containing 1 g of poloxamer formulations were left for 10 min in a water bath of RT or 37 °C in order to allow the samples to equilibrate thermally to the bath temperature. After thermal equilibration, the sample vials were removed from the water bath and were inverted immediately to observe the sample flow in the vials. When the sample remained at the bottom of the vial after inversion the status of these samples were recorded as the gel. All experiments were performed in triplicate.

Table S1. Rheological properties of P407 solutions or gels containing various concentrations of propolis extracts.

P407 (%)	Propolis (%)	4°C	RT	37°C
15	0	Sol	Sol	Gel
	5	Sol	Gel	Gel
	10	Gel	Gel	Gel
20	0	Sol	Sol	Gel
	6	Sol	Gel	Gel
	10	Gel	Gel	Gel
	20	Gel	Gel	Gel

Supplement 3. Antimicrobial activities of propolis without poloxamer and vehicle controls of 5% and 10% P188 solutions

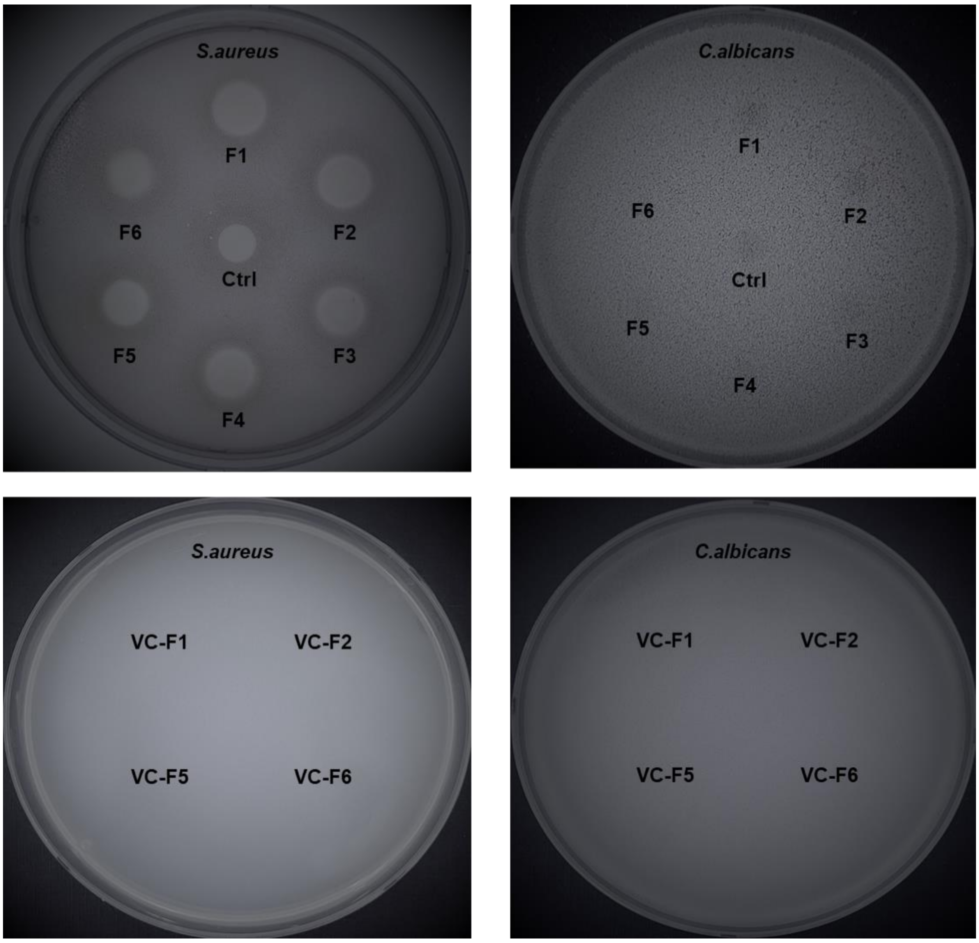

Figure S3. Antimicrobial susceptibility of propolis without poloxamers and vehicle controls. In both *S. aureus* and *C. albicans*, the antimicrobial susceptibility of the formulations was superior to that of the control (Ctrl) without poloxamer (top panels). The vehicle controls (VC) without propolis did not show any effect under the conditions of the antimicrobial susceptibility test (bottom panels). .

Supplement 4. AUC of time-kill kinetics of *S. aureus* and C. *albicans* in VC of P188 and P407 formulations without propolis.

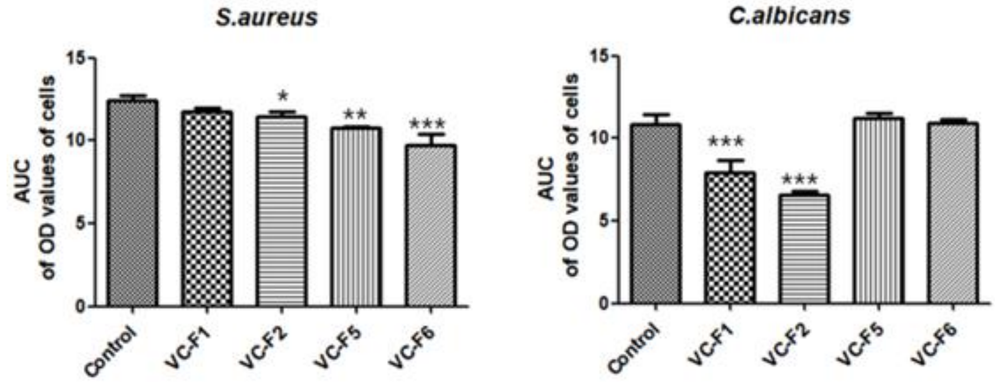

Figure S4. AUC of time-kill kinetics of *S. aureus* and *C. albicans* in VC of P188 and P407 formulations without propolis. Data are presents as mean ± SD. (n=3) ($*p < 0.05$, $**p < 0.01$, $***p < 0.001$ compared to the control, one-way ANOVA followed by Dunnett's post hoc test)

Supplement 5. Antimicrobial activities of propolis, propylene glycol-extract and ethanol-extract formulated in P188 and P407

Experimental methods.

Antimicrobial kinetics assay

For antimicrobial kinetics assay, cells were cultured to OD_{600} of 1.0 in LB or YM broth and diluted to approximately 1 x 10^6 CFU/ml suspension. Two different sources of propolis, propylene glycol-extract and ethanol-extract were formulated with P188 and P407 at 5 and 10% concentration and diluted in broth. They were then mixed with the cell suspension at a ratio of 1:1. Each mixture (100 μl) was inoculated into a 96-well microtiter plate. Ethanol-extract propolis showed a superior antimicrobial effect than propylene glycol-extract and therefore, was diluted 4-fold more than in Fig 6. to compare the effects according to poloxamer type for each extract. The final concentration of propolis in each well was 625 μg/ml. Unformulated propolis ethanol-extract was dissolved in 10% DMSO. A separate experiment confirmed that the DMSO at this concentration did not affect the cells. Inoculated 96-well plates were incubated for 18 hr at 37 °C for *S. aureus* and 30 °C for *C. albicans*, while measuring their OD_{600} every 20-minute using an Epoch™ 2 Microplate Spectrophotometer (BioTek Inc., USA).
